# Oral Capsaicinoid Administration Alters the Plasma Endocannabinoidome and Fecal Microbiota of Reproductive-Aged Women Living with Overweight and Obesity

**DOI:** 10.3390/biomedicines9091246

**Published:** 2021-09-17

**Authors:** Claudia Manca, Sébastien Lacroix, Francine Pérusse, Nicolas Flamand, Yvon Chagnon, Vicky Drapeau, Angelo Tremblay, Vincenzo Di Marzo, Cristoforo Silvestri

**Affiliations:** 1 Département de Médecine, Faculté de Médecine, Université Laval, Québec, QC G1V 0A6, Canada; claumanca@unica.it (C.M.); nicolas.flamand@criucpq.ulaval.ca (N.F.); yvon-c.chagnon.1@ulaval.ca (Y.C.); 2 Canada Excellence Research Chair on the Microbiome-Endocannabinoidome Axis in Metabolic Health, Québec, QC G1V 4G5, Canada; sebastien.lacroix.8@ulaval.ca; 3 Institut Universitaire de Cardiologie et de Pneumologie de Québec, Université Laval, Québec, QC G1V 4G5, Canada; vicky.drapeau@fse.ulaval.ca (V.D.); angelo.tremblay@kin.ulaval.ca (A.T.); 4 Nutrition, Santé et Société (NUTRISS), Institut sur la Nutrition et les Aliments Fonctionnels (INAF), Université Laval, Québec, QC G1V 0A6, Canada; francine.perusse@kin.ulaval.ca; 5 Département de Kinésiologie, Faculté de Médecine, Université Laval, Québec, QC G1V 0A6, Canada; 6 Département d’éducation Physique, Faculté des Sciences de l’éducation, Université Laval, Québec, QC G1V 0A6, Canada; 7 Faculté des Sciences de l’Agriculture et de l’Alimentation, Université Laval, Québec, QC G1V 0A6, Canada; 8 Institute of Biomolecular Chemistry, National Research Council, 80078 Pozzuoli, Italy

**Keywords:** capsaicinoids, endocannabinoidome, microbiota, overweight, obesity, food intake, lipidomics, metabolism

## Abstract

Capsaicinoids, the pungent principles of chili peppers and prototypical activators of the transient receptor potential of the vanilloid type-1 (TRPV1) channel, which is a member of the expanded endocannabinoid system known as the endocannabinoidome (eCBome), counteract food intake and obesity. In this exploratory study, we examined the blood and stools from a subset of the participants in a cohort of reproductive-aged women with overweight/obesity who underwent a 12-week caloric restriction of 500 kcal/day with the administration of capsaicinoids (two capsules containing 100 mg of a capsicum annuum extract (CAE) each for a daily dose of 4 mg of capsaicinoids) or a placebo. Samples were collected immediately before and after the intervention, and plasma eCBome mediator levels (from 23 participants in total, 13 placebo and 10 CAE) and fecal microbiota taxa (from 15 participants in total, 9 placebo and 6 CAE) were profiled using LC–MS/MS and 16S metagenomic sequencing, respectively. CAE prevented the reduced caloric-intake-induced decrease in beneficial eCBome mediators, i.e., the TRPV1, GPR119 and/or PPARα agonists, *N*-oleoyl-ethanolamine, *N*-linoleoyl-ethanolamine and 2-oleoyl-glycerol, as well as the anti-inflammatory *N*-acyl-ethanolamines *N*-docosapentaenyl-ethanolamine and *N*-docosahexaenoyl-ethanolamine. CAE produced few but important alterations in the fecal microbiota, such as an increased relative abundance of the genus *Flavonifractor*, which is known to be inversely associated with obesity. Correlations between eCBome mediators and other potentially beneficial taxa were also observed, thus reinforcing the hypothesis of the existence of a link between the eCBome and the gut microbiome in obesity.

## 1. Introduction

Non-communicable diseases, such as heart disease, stroke, cancer, chronic respiratory diseases and diabetes, are the leading causes of mortality in the world [1]. Among the common, modifiable risk factors, such as alcohol consumption, unhealthy diets, insufficient physical activity, increased blood pressure, blood sugar and cholesterol, obesity represents a growing major public health problem in most countries, with an incidence that has nearly tripled since 1975 [2]. As such, obesity (particularly visceral obesity in subjects living with overweight) and the related comorbidities impose an enormous burden at the individual, public health and economic levels [3].

Notwithstanding the idea that some nutrients are more obesogenic than others, overweight and obesity occur when energy intake exceeds energy expenditure, causing a state of positive energy balance and a consequent increase in body mass, of which 60–80 percent is usually body fat [4]. Since a positive energy balance is a problem for many people in Western countries, a healthy lifestyle, including a balanced diet, is necessary to prevent or reverse an excess energy intake over expenditure. However, due to the multifactorial nature of obesity—encompassing hereditary factors, ethnic differences, environment and individual behavior—efficient strategies to regulate energy intake that lead to significant and sustained weight loss are not well defined, and, to date, no clear non-surgical solution has been developed [4,5]. Indeed, pharmaceutical drugs, such as orlistat, which is a potent and specific inhibitor of intestinal lipases that can reduce body weight with variable efficacy, can lead to gastrointestinal, hepatic and kidney injuries [6,7]. Recently, the glucagon-like peptide-1 analog semaglutide, in conjunction with calorie restriction, induced significantly more weight loss than a placebo [8]. Other anti-obesity drugs were withdrawn from the market due to severe adverse effects, including increased cardiovascular risks, mood disorders and even suicidal susceptibility [9]. Increasing efforts are therefore aimed at developing novel strategies, including active natural ingredients and functional foods, which could facilitate appetite control, to promote favorable changes in body energy stores and exert long-term benefits on metabolic health [6].

Among a plethora of identified natural anti-obesity compounds, such as celastrol (from the roots of the thunder god vine) [10], the stilbenoid resveratrol (from grapes and red wine), genistein (an isoflavone from soy), glycyrrhizin (from licorice), quercetin, ethanolic extracts from ginseng roots and green tea extract [11], chili pepper, with its more than 200 active constituents, was suggested to display energy-expenditure-stimulating, anorexigenic, anti-hypertensive, vasodilatory, cardioprotective and, finally, anti-obesity actions [12]. In particular, capsaicin, a non-nutritional food constituent and the main pungent principle in hot red peppers and its major active compound, seems to have multiple beneficial effects in the treatment of pain, inflammation, cancer and oxidative stress, among others [12,13,14,15,16,17], and was suggested as an anti-obesity therapeutic [18]. Capsaicin was observed to decrease appetite and subsequent protein and fat intake in Japanese females and to lower energy intake in Caucasian males [19]. These effects were potentially related to an increase in sympathetic nervous system (SNS) activity. This was in agreement with the observation that pre-prandial intake of capsaicin, be it in capsules or diluted in tomato juice, increases satiety, thus reducing energy intake [20]. Capsaicin was also found to decrease energy intake when tested in combination with caffeine [21]. Epidemiological evidence suggested that the incidence of obesity is lower in individuals who consume capsaicinoid-containing foods [22]. Furthermore, post-weight loss fat oxidation following a 4-week very low energy dietary intervention was better maintained over 3 months by individuals that were supplemented with capsaicin [23]. Finally, a recent meta-analysis of controlled human trials evidenced how dietary capsaicinoids produce beneficial effects on metabolic parameters, such as serum total cholesterol levels, also in non-obese subjects and independently from effects on BMI and body fat [24].

Capsaicin’s positive metabolic effects appear to be due, at least in part, to the modulation of the transient receptor potential vanilloid subtype 1 (TRPV1) channel, which plays a critical role in the regulation of metabolic health, including body weight, glucose and lipid metabolism, white and brown adipose tissue biology and the cardiovascular system [25,26]. It was demonstrated that the activation of TRPV1 by capsaicin can attenuate abnormal glucose homeostasis by stimulating insulin secretion and increase glucagon-like peptide 1 (GLP-1) levels [27,28]. Although it is well accepted that much of the effect is caused by stimulation of the TRPV1 receptor, other mechanisms of action were described. Capsaicin can indeed play its role in a receptor-independent manner through the inactivation of nuclear factor κB (NF-κB) and activation of peroxisome proliferator-activated receptor γ (PPARγ) [29]. Another mechanism is represented by the regulation of the production of the endocannabinoids (eCBs), which are lipid mediators that act as major regulators of energy homeostasis [30,31,32] and, together with a plethora of eCB-related molecules, receptors and metabolic enzymes, constitute the endocannabinoidome (eCBome), a complex lipid-signaling system [33]. Some of these molecules, such as long-chain unsaturated mono-acyl-glycerols (MAGs) and, particularly, *N*-acyl-ethanolamines (NAEs), are well-established endogenous activators of TRPV1 channels [34,35]. Interestingly, the eCBome is increasingly understood to communicate with the gut microbiome in the context of metabolic disorders [36,37,38]. Similarly, capsaicin modulates the gut microbiota population, which represents a critical factor for the anti-obesity effects that are exerted by this natural compound, contributing to improving glucose homeostasis through increasing short-chain fatty acids, regulating gastrointestinal hormones and inhibiting pro-inflammatory cytokines [39,40]. A very recent study suggested that such effects might be at least in part mediated by the activation of TRPV1 since the ablation of this ligand-activated channel from the rat intestine was found to cause gut dysbiosis, as evidenced by the decreased abundance of beneficial taxa and production of short-chain fatty acids, and by the impairment of colonic mucus secretion [41].

These elements prompted us to investigate the impact of the intake of capsaicinoids (i.e., capsaicin and its TRPV1-active analogs that are present in *Capsicum annuum*) on the plasma eCBome and gut microbiome in individuals with overweight/obesity. For this purpose, we took advantage of the availability of a limited number of blood and fecal samples from a clinical trial where the effect of a combination of dietary capsaicin (provided in the form of *Capsicum annuum* extract (CAE) capsules) with a low-caloric intervention was tested on appetite sensations, energy intake and expenditure, body weight and fat distribution in free-living, physically non-trained reproductive-aged women with overweight and obesity.

## 2. Materials and Methods

### 2.1. Ethical Approval

This study was conducted according to the guidelines laid down in the Declaration of Helsinki and all procedures involving human subjects were approved by the Research Ethics Committee of Health Sciences of Laval University (protocol 2015-041 A-4 R-2/01-05-2018). Written informed consent was obtained from all the participants before entering the study. The clinical trial was registered in the public trials registry ClinicalTrials.gov (registration identification number NCT04874701).

### 2.2. Subjects and Treatment Protocol

Sixty-one reproductive-aged women with overweight or obesity (25 kg/m^2^ ≤ BMI ≤ 35 kg/m^2^) aged between 18 and 50 years had their health status assessed by a physician to confirm their eligibility against inclusion/exclusion criteria. Eligible volunteers could not be pregnant, had to have stable body weight, needed to be free from chronic diseases, could not take any medication or have restrictive dietary habits or food allergies. Sixty-one volunteers were enrolled and underwent a first session (visit 1 (V1)), during which overnight-fasted participants were assessed for their resting metabolic rate and substrate oxidation. Blood and stool samples were collected, preferably before breakfast. Volunteers were randomly distributed into two groups: the CAE group (30 participants that took two CAE capsules per day (100 mg *Capsicum annuum*/capsule containing 2% capsaicinoids; 1.2% capsaicin, 0.7% dihydrocapsaicin and 0.1% nordihydrocapsaicin; OmniActive Health Technologies)) and the placebo group (31 participants). The capsules were taken at breakfast. The composition of CAE and placebo capsules are detailed in Table 1.

Each participant received a 12-week personalized dietary plan with a 500 kcal/day energy restriction (RMR × physical activity level − 500 kcal). Specifically, in order to respect the restriction, the volunteers were provided with a precise meal plan. Every two weeks, the participants met the dietitian in order to ensure adequate compliance with the prescribed diet, to measure their heart rate and blood pressure, to verify the adequacy of capsule consumption and to provide motivational support. After the 12 weeks of supplementation (visit 7 (V7)), the subjects participated in a second testing session that was the same as the first one. Body composition (body fat and fat-free mass) was measured using dual X-ray absorptiometry at the beginning and the end of the program. Seventeen participants did not complete the study, resulting in a dropout rate of 27%. Furthermore, one subject displayed a body weight gain of 8 kg, which was considered as an unacceptable outlier from a clinical and a statistical standpoint and was removed from the analysis. Twenty-two placebo- and 21 CAE-treated women completed the study. However, only a limited subset of participants could be subjected to an assessment of fecal microbiota composition (15 participants in total, 9 placebo and 6 CAE) and plasma eCBome mediator levels (23 participants in total, 13 placebo and 10 CAE) at V1 and V7 due to the fact that such measurements were introduced in the protocol only after the start of the study (see Table S1 for an overview of CAE and placebo samples).

### 2.3. Assessment of Anthropometric Parameters

Height, weight and waist circumference were measured at baseline and every two weeks over the course of the intervention. Height measurements were performed using a Stadiometer Holtain. Body weight was measured with a Tanita Body Composition Monitor scale and waist circumference (located between the bottom of the ribs and the iliac crest) was measured using a tape graduated in centimeters.

### 2.4. Measurement of Appetite Sensations and Eating Behaviors

Appetite sensations (hunger, desire to eat, fullness, prospective food consumption) were assessed using a visual analog scale (VAS) before a standardized breakfast containing 599 kcal was offered using the procedures of Drapeau et al. [42]. Participants then received two CAE capsules or a placebo and were then monitored for appetite sensations using the VAS, calorimetry measurements and blood pressure every 30 min during a 180-min period. After 3.5 h, participants were offered a lunch buffet that was used to measure ad libitum energy and macronutrient intake [43]. The VAS was used to measure appetite every 30 min during a 240 min period following consumption of the meal. At the end of the afternoon, the participants were invited to consume an ad libitum standardized dinner. Appetite and blood pressure were measured before and immediately after consumption of the meal. These procedures were repeated on the last visit following the 12-week intervention period.

As for the first testing session of the protocol, which assessed the effects of a standardized breakfast on post-prandial appetite sensations, satiety was measured as a score of post-prandial appetite feelings. As for the second testing session, which was aimed at evaluating the buffet-type meal served at lunchtime, as well as variations in appetite sensations in the afternoon and energy/macronutrient intake at dinner time, satiety was mostly measured as the ad libitum energy intake at lunchtime. Since the ad libitum energy intake was not the same for each subject, we calculated the satiety quotient, which was determined as the change in afternoon appetite sensations relative to the ad libitum energy intake at lunchtime [42,44]. These observations were completed using the measurement of energy intake at dinner time, which provided a more complete outcome on cumulative energy intake at a time that CAE was entirely made available.

### 2.5. Measurement of Eating Behavior

Eating behaviors were assessed at baseline and at 12 weeks with the 51-item Three-Factor Eating Questionnaire (TFEQ), which measures habitual cognitive restraint, disinhibition and susceptibility for hunger [45], and with the Food Cravings Questionnaire [46].

### 2.6. Lipid Extraction and HPLC–MS/MS for the Analysis of eCBome Mediators

eCBome mediators were extracted from V1 and V7 plasma samples of a total of 23 participants (13 placebo and 10 CAE). Sample processing and LC–MS/MS parameters were exactly as described by Turcotte and collaborators [47]. The quantification of eCBome-related mediators (Table S2) was carried out by an HPLC system interfaced with the electrospray source of a Shimadzu 8050 triple quadrupole mass spectrometer and using multiple reaction monitoring in positive ion mode for the compounds and their deuterated homologs.

In the case of unsaturated monoacylglycerols, the data are presented as 1/2-monoacylglycerols (2-MAGs) but represent the combined signals from the 2- and 1(3)-isomers since the latter were most likely generated from the former via acyl migration from the *sn*-2 to the *sn*-1 or *sn*-3 position.

### 2.7. DNA Extraction and 16S rRNA Gene Sequencing

DNA was extracted from the V1 and V7 fecal samples of a total of 18 participants (12 placebo and 6 CAE) using the QIAmp PowerFecal DNA kit (Qiagen, Hilden, Germany) according to the manufacturers’ instructions. The DNA concentrations of the extracts were measured fluorometrically with the Quant-iT PicoGreen dsDNA Kit (Thermo Fisher Scientific, Waltham, MA, USA) and the DNA samples were stored at −20 °C until the 16S rDNA library preparation. Briefly, 1 ng of DNA was used as a template and the V3–V4 region of the 16S rRNA gene was amplified by polymerase chain reaction (PCR) using the QIAseq 16S Region Panel protocol in conjunction with the QIAseq 16S/ITS 384-Index I (Sets A, B, C, D) kit (Qiagen, Hilden, Germany). The 16S metagenomic libraries were eluted in 30 µL of nuclease-free water and 1 µL was qualified with a Bioanalyser DNA 1000 Chip (Agilent, Santa Clara, CA, USA) to verify the amplicon size (expected size ~600 bp) and quantified with a Qubit (Thermo Fisher Scientific, Waltham, MA, USA). Libraries were then normalized and pooled to 4 nM, denatured and diluted to a final concentration of 10 pM and supplemented with 5% PhiX control (Illumina, San Diego, CA, USA). Sequencing (2 × 300 bp paired-end) was performed using the MiSeq Reagent Kit V3 (600 cycles) on an Illumina MiSeq System. Data were processed using the DADA2 pipeline [48] and assignation to bacterial taxa was obtained using the Silva v132 reference database. Sequences that were present in fewer than 3 samples were filtered out and bacterial abundances were normalized using the Cumulative Sum Scaling (CSS, *MetagenomeSeq* R package) [49]. Alpha-diversity indices were obtained using the *Phyloseq* R package.

### 2.8. Statistical Analyses

Values are presented as mean ± standard error. The effects of interventions on eCBome mediators were assessed using Student’s *t*-test comparing the pre- and post-intervention (i.e., visit 1 (V1) and visit 7 (V7), respectively) levels, for placebo and CAE individually. Post-intervention levels were also compared between placebo and CAE groups. Variations in fecal microbiota compositions were evaluated using two-way analysis of variance (ANOVA) and Tukey’s HSD post hoc tests to assess the treatment (placebo vs. CAE), time (before vs. after dietary intervention, i.e., visit 1 (V1) and visit 7 (V7), respectively) and treatment × time interactions. Correlations between the eCBome mediators, microbial taxa, anthropometric parameters and appetite sensations were obtained using repeated measures correlation tests (*rmcorr* package), which accounted for the within-individual relationships between paired measures [50]. A *p*-value of 0.05 was selected as reflecting a statistically significant effect.

## 3. Results

### 3.1. Trial Completion and Side Effects

As shown in Table S1, 43 participants completed the trial and 17 dropped out of the study. Out of the 61 participants, 39 (14 placebo and 25 CAE) reported symptoms. Table S1 presents the list of symptoms reported by the two groups of participants. Most side-effects were minor and related to gastrointestinal disturbance and were more frequent in the CAE group. One subject experienced an adverse event that necessitated hospitalization. Of the 17 participants that dropped out of the study (6 placebo and 11 CAE), only three did so because of side effects, whilst the other dropouts were due to personal reasons.

### 3.2. Effect of CAE and Caloric Restriction on Body Weight, BMI, Waist Circumference and Fat Mass

Full details of the effects of CAE on these endpoints, which were assessed in the whole cohort, will be reported elsewhere. For the purpose of this article, we believe that it will suffice to state that caloric restriction, alone or supplemented with CAE, induced significant reductions in body weight, BMI, waist circumference and fat mass percentage from baseline to V7. Reductions of anthropometric variables associated with CAE were non-significantly different than for the placebo treatment: body weight (↓ 3.81% vs. ↓ 3.08%), BMI (↓ 3.97% vs. ↓ 2.34%), waist circumference (↓ 2.83% vs. ↓ 2.18%) and fat mass percentage (↓ 4.03% vs. ↓ 3.20%), though trends toward significance were observed for decreased body weight (*p* = 0.1) and fat-free mass (*p* = 0.08) (Table 2).

More importantly for the purpose of this article, in the subset of patients whose plasma eCBome mediator levels and fecal microbiota composition were determined, waist circumferences were reduced in both the placebo and CAE groups (↓ 1.98 ± 0.65 and ↓ 1.45 ± 0.77, respectively; *p* = 0.009 vs. V1 for both), while body weight and BMI were non-significantly different from baseline. Interestingly, only within the CAE group was the fat mass percentage was reduced (↓ 3.73 ± 1.25, *p* = 0.01) and fat-free mass increased (↑ 2.47 ± 0.79, *p* = 0.01).

### 3.3. Effect of CAE and Caloric Restriction on Measures of Appetite Sensation and Eating Behavior

Full details of the effects of CAE on these endpoints, which were assessed in the whole cohort, will be reported elsewhere. For the purpose of this article, we believe that it will suffice to state that caloric-restriction-induced weight loss caused an increase in self-administered VAS-derived desire to eat, hunger and prospective food consumption. However, no additional effect could be observed with CAE (Table 3). No significant effect of either caloric restriction alone or supplementation with CAE was observed using the TFEQ hunger subscale (TFEQ-HUN) (Table 4).

We then sought to investigate whether these observations could also be reported in the subset of participants that had both their plasma eCBome and fecal microbiota composition characterized (Table 5). Pre-meal evaluation of the amount of food that could be eaten (DQuaN) was drastically increased by weight loss that was either induced by caloric restriction alone or in conjunction with CAE supplementation. The pre-meal feeling of hunger (BSenF) increased during caloric restriction-induced weight loss (placebo V1 vs. V7). The CAE supplementation did not seem to influence any subjective measures of hunger or satiety. Conversely, susceptibility to hunger, as derived from the TFEQ hunger subscale (TFEQ-HUN) was reduced by caloric restriction only (placebo V1 vs. V7), which was an effect that seemed to be due to reduced feelings of hunger arising from internal sources (TFEQ-HIL).

### 3.4. Effect of CAE and Caloric Restriction on Plasma ECBome Mediator Levels

**Figure 1:**
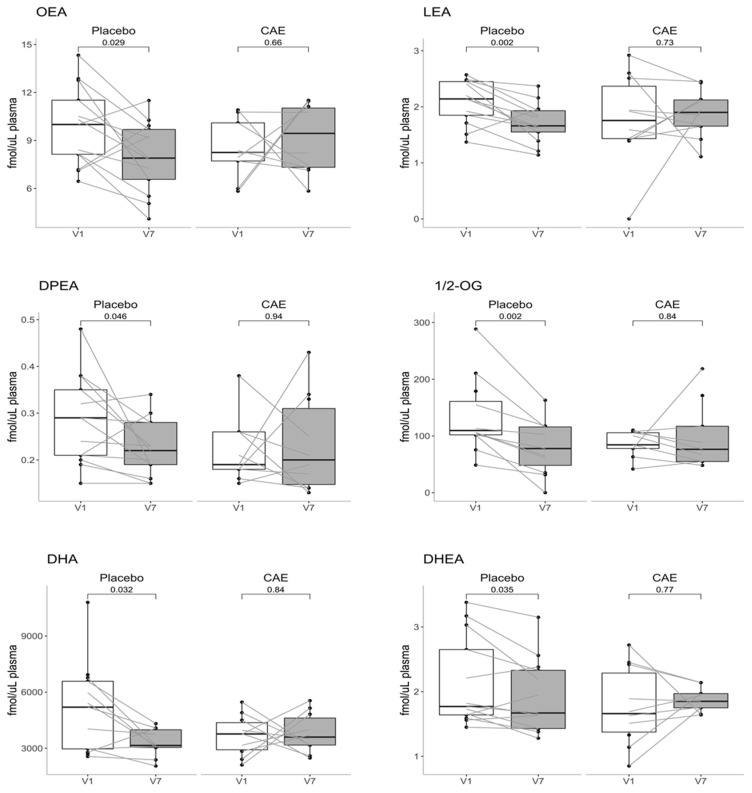
Effect of caloric restriction without or with CAE on plasma eCBome mediator levels. eCBome mediator levels in the plasma of a subset of 23 participants (13 placebo and 10 CAE) before (visit 1 (V1)) and after (visit 7 (V7)) the interventions. The levels of the eCBome-related mediators measured using HPLC-MS/MS are expressed in femtomoles per microlitre of plasma. Each median is represented by the middle line of each boxplot, Q1 and Q3 are represented by the bottom and top of the boxes, respectively, and whiskers denote 1.5 × IQR. OEA, *N*-oleoyl-ethanolamine; LEA, *N*-linoleoyl-ethanolamine; DPEA, *N*-docosapentaenoyl-ethanolamine; 1/2-OG, 1/2-oleoyl-glycerol; DHA, docosahexaenoic acid; DHEA, *N*-docosahexaenoyl-ethanolamine.

As mentioned above, this endpoint could be measured only in a relatively small subset of the participants from the larger study (i.e., 13 placebo and 10 CAE); nevertheless, its assessment was one of the two major objectives of this article. Caloric restriction alone (placebo V1 vs. V7) induced important reductions in the plasma levels of *N*-oleoyl-ethanolamine (OEA), *N*-oleoyl-ethanolamine (LEA), *N*-docosapentaenoyl-ethanolamine (DPEA) and 1,2-oleoyl-glycerol (1/2-OG), as well as in the levels of the omega-3 fatty acid docosahexaenoic acid and its eCBome derivative *N*-docosahexaenoyl-ethanolamine (DHEA) (Figure 1). However, the addition of CAE prevented all these decreases.

### 3.5. Effect of CAE and Caloric Restriction on Fecal Microbiota Composition

**Figure 2:**
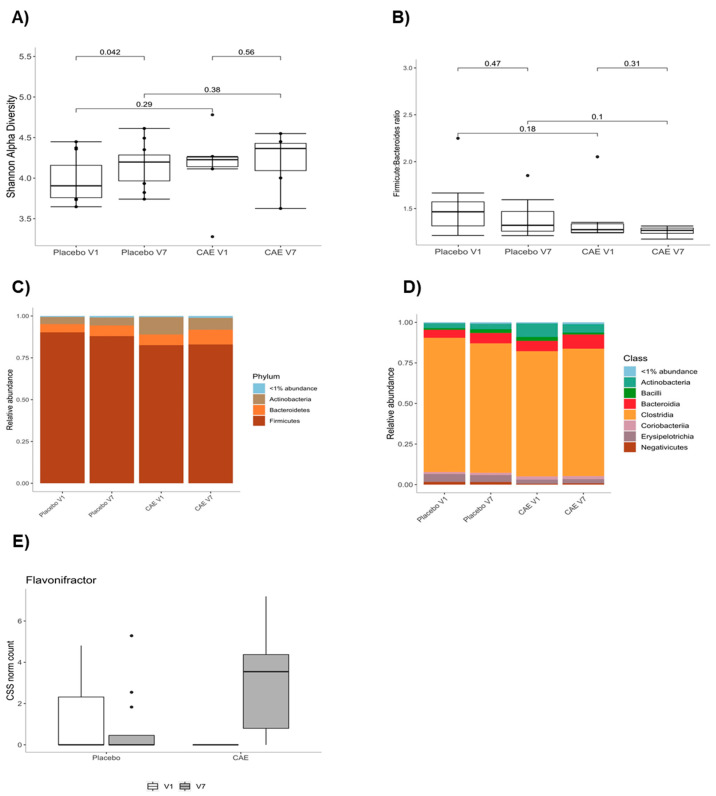
Effect of caloric restriction without or with CAE on the fecal microbiota composition. Alpha diversity of the fecal microbiota (**A**), Firmicutes:Bacteriodetes ratio (**B**) and the relative abundances of fecal microbiota phyla (**C**), classes (**D**) and the Flavonifractor genera (**E**). The data were obtained from stool samples of a subset of 25 (19 placebo and 6 CAE) reproductive-aged women with overweight or obesity before (visit 1 (V1)) and after (visit 7 (V7)) the interventions.

As mentioned above, this endpoint could be measured only in a relatively small subset of the participants to the larger study (i.e., 9 placebo and 6 CAE), where its assessment was the other major objective of this article. Caloric restriction caused an increase in the alpha diversity of the fecal microbiota at V7, which was, however, counteracted by CAE administration, possibly also due to a trending difference between the V1 placebo and V1 CAE groups (Figure 2A). The Firmicutes/Bacteriodetes ratio was not significantly reduced following caloric restriction, either in the presence or absence of CAE (Figure 2B). Accordingly, no changes in the relative abundance of fecal microbiota phyla were observed following caloric restriction. However, a trend (*p* = 0.07) toward increased Bacteroidetes was observed following CAE administration (Figure 2C). At the class level, Clostridia (*p* = 0.043) and Actinobacteria (*p* = 0.021) were increased at visit 7 with CAE as compared to the placebo controls (Figure 2D). Finally, and perhaps more importantly, at the genus level, Flavonifractor was increased by CAE over the course of the treatment but not by caloric restriction alone (Figure 2E). However, Faecalibacterium, Ruminococcaceae_UCG-004 and Ruminococcaceae_UCG-010 showed significant differences in their relative abundances at baseline (V1) between placebo and CAE groups, which may have obfuscated CAE-induced effects (Figure S1). Nevertheless, Faecalibacterium showed a trend toward significance (*p* = 0.14) for increased abundance in response to CAE treatment (V1 vs. V7).

### 3.6. Correlations between Plasma ECBome Mediator Levels, Anthropometric Measures and Fecal Microbiota Composition

**Figure 3:**
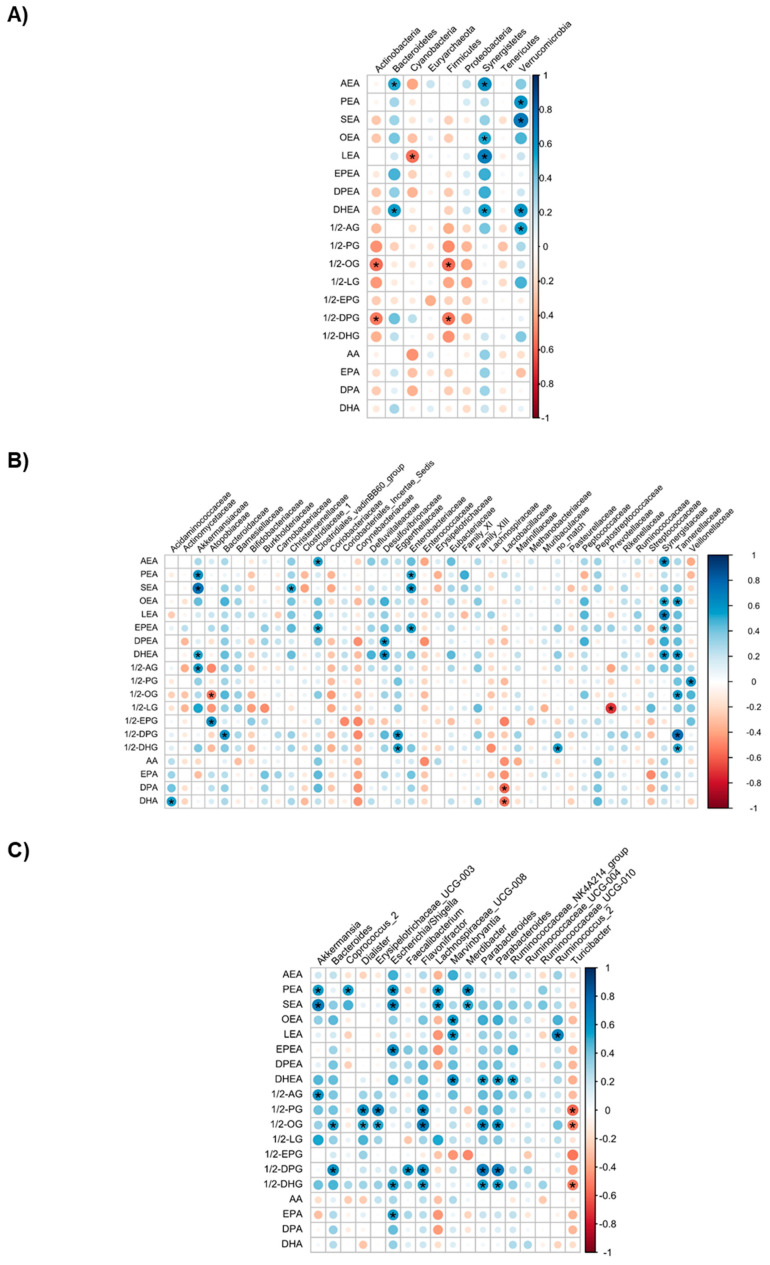
Correlations between the plasma levels of eCBome mediators and the relative abundance of phyla (**A**), families (**B**) and genera (**C**) of the fecal microbiota of 15 (9 placebo and 6 CAE) reproductive-aged women with overweight or obesity. The correlation analysis was performed using repeated measures correlation tests (rmcorr package). * indicates a correlation coefficient with *p*-value < 0.05.

Next, we assessed whether any correlation existed between the plasma levels of eCBome mediators and other outcome measures within individuals for which the appropriate data were collected. Several eCBome mediators correlated with the relative abundances of the phyla, families and genera of the fecal microbiota (Figure 3). In particular, among the pyla, MAGs appeared to correlate negatively with Actinobacteria and Firmicutes (in the case of 1/2-OG and 1/2-DPG the correlations were statistically significant), whereas NAEs correlated positively with Bacteroidetes (significantly for the endocannabinoid anandamide (AEA) and for DHEA), Synergistetes (significantly for AEA, OEA, LEA and DHEA) and Verrucomicrobia (significantly for DHEA, *N*-palmitoyl-ethanolamine (PEA) and *N*-stearoyl-ethanolamine (SEA)). Significant lipid-class-independent correlations were also found with some phyla, i.e., for Cyanobacteria (negative), which was only significantly correlated with the LEA levels, and Verrucomicrobia (positive), which was significantly correlated with the endocannabinoid 2-arachidonoyl-glycerol (2-AG) (calculated together with its 1-isomer (1/2-AG)) and several NAEs (Figure 3A). Stronger correlations were found at the family and genus levels (Figure 3B,C). Most relevant correlations, namely, those that occurred between the eCBome mediators that were found here to be differentially affected by caloric restriction and CAE treatment (Figure 1), include: (1) At the family level, Akkermansiaceae positively correlated with DHEA; Desulfovibrionaceae with DPEA and DHEA; Synergistaceae with OEA, LEA, DPEA and DHEA; and Tannerellaceae with OEA, DHEA and 2-OG. Meanwhile, Atopobiaceae and Prevotellaceae correlated negatively with 2-OG. (2) At the genus level, Akkermansia, Coprococcus 2, Escherichia/Shigetta, Lachnospiraceae UCG-008 and Merdibacter positively correlated with PEA, Bacteroides, Erysipelotrichaceae UCG003, Dialister, Flavonifractor, Parabacteroides and 2-OG; Marvinbryantia with OEA and DHEA; Marvinbryantia and Ruminococcus 2 with LEA; Parabacteroides and the Ruminococcaceae NK4A214 group with DHEA; and Turicibacter with 2-OG.

We also found significant correlations between several fecal microbiota taxa and body weight, BMI and fat mass (FM) (Figure S2). Interestingly, families that negatively correlated with BMI, weight and FM were positively correlated with fat-free mass (FFM) (even when only non-statistically significant trends were observed and vice versa). Therefore, some of the above-mentioned correlations might, at least in part, be mediated by variations of these anthropometric measures. However, of the taxa mentioned above, only the Akkermansia genus (from the Verrucomicrobia phylum and Akkermansiaceae family) was correlated (negatively) with body weight, BMI and fat mass (Figure S2C), and not with the eCBome mediators that correlated with these parameters (except for 1/2-AG). This suggests that these correlations were independent of the variations in these anthropometric measures. Conversely, the positive correlations between Akkermansia (and associated phylogenic levels) and 1/2-AG levels (Figure 3B) suggest that this mediator correlated negatively with BMI and body weight (Figure S3).

### 3.7. Correlations between Plasma ECBome Mediator Levels and Appetite Sensations and Eating Behaviors

**Figure 4:**
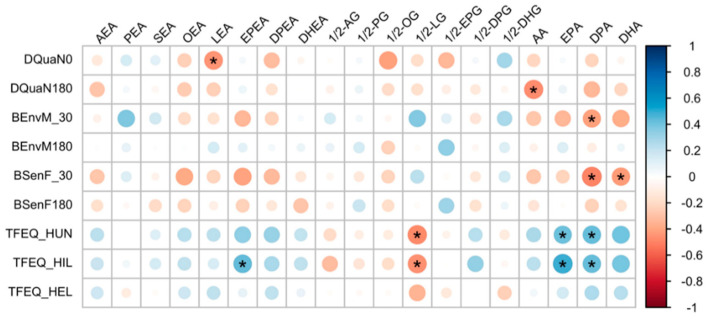
Correlations between the plasma levels of eCBome mediators and the appetite and satiety measures of 15 (9 placebo and 6 CAE) reproductive-aged women with overweight or obesity. The correlation analysis was performed using repeated measures correlation tests (rmcorr package). Only correlation coefficients with *p*-value < 0.05 are displayed. DQuaN, pre-meal evaluation of the amount of food that could be eaten; BEnvM, pre-meal desire to eat; BSenF, feeling of hunger; TFEQ-HUN, habitual feelings of hunger; TFEQ-HIL, feelings of hunger arising from internal sources. * indicates a correlation coefficient with *p*-value < 0.05.

Our investigation of the associations between plasma eCBome mediator levels and appetite and satiety measures were motivated by previously reported roles of some eCBome mediators and receptors on regulating food intake [51] (Figure 4). The amount of food that could be eaten (DQuaN) was inversely related to levels of LEA, while pre-meal desire to eat (BEnvM) and feeling of hunger (BSenF) were inversely related to DPA and both DPA and DHA, respectively.

A habitual feeling of hunger (TFEQ-HUN) was inversely related to circulating levels of 1/2-LG and subscale of hunger and hunger from internal (e.g., affect, arousal or stress) cues (TFEQ-HUN and HIL) were positively correlated to the levels of long-chain omega-3 precursors EPA and DPA. Conversely, hunger from external food cues, which are mostly related to situations or objects that have previously been associated with food intake, did not show any correlation with the mediators analyzed.

## 4. Discussion

The main novel result of this exploratory study is that a capsaicinoid-based supplement produced effects on the eCBome and the gut microbiota, potentially facilitating an amelioration of the metabolic status in women with overweight and obesity. These effects may represent early adaptations that could ultimately result in beneficial effects on food intake, body weight and fat accumulation, as possibly suggested by the present finding showing that, in the subset of patients that underwent plasma eCBome and gut microbiota assessment, CAE performed better than the placebo at enhancing caloric-restriction-induced fat mass reduction. However, in the whole cohort (whose plasma eCBome and fecal microbiome could not be entirely assessed for logistical reasons), CAE enhanced the effects of caloric restriction on body weight, waist circumference and fat mass only in a non-statistically significant manner. It is possible that either a more or a less pronounced caloric-restriction-induced weight/fat loss must be achieved in order to allow for the beneficial effects on the eCBome and gut microbiota that are induced by CAE to produce a stronger impact also on these two anthropometric measures. Indeed, previous studies showed that the same CAE used here, under the same dosing protocol, does produce reductions in body weight and fat mass in a larger cohort of both male and female individuals with lower starting BMI and in the absence of concomitant caloric restriction [52,53]. Future clinical studies, as well as a more thorough examination of the data from the whole cohort than that performed in this article, which was focused on the effects of CAE on the plasma eCBome and fecal microbiota composition, need to be awaited in order to draw any definitive conclusion as to what extent these natural products can be used as a therapy against obesity and hyperphagia.

Indeed, capsaicin is one of the most studied natural products and food (spice) components in the context of metabolic disorders and related cardiovascular risk [3,54,55]. Epidemiological studies suggested that hot chili pepper consumption may positively affect some parameters of the metabolic syndrome, particularly hypertension and body fat [12,56]. A meta-analysis of clinical studies supported the notion that, especially at high doses, capsaicin positively affects energy expenditure and appetite regulation, though contradictory results were often observed in studies with subjects living with overweight and obesity [57]. Several preclinical studies with oral capsaicin supported the possible use of this compound to treat metabolic disorders, also due to effects on food intake, satiety and energy expenditure [28,58,59]. In some of these animal studies, capsaicin effects were ascribed, at least in part, to its capability of modifying the composition of the gut microbiota, for example, in a manner that enhances short-chain fatty acid-producing bacteria [39,40,60]. In one case, these beneficial and gut-microbiota-mediated effects of capsaicin were shown to be due to counteracting the CB1 overactivation by endocannabinoids in obesity [39]. However, in only a few of these experimental studies, the role of TRPV1, which is the major target for capsaicin, was investigated and demonstrated [41], despite the fact that also this ligand-activated channel is known to be strongly implicated in several aspects of energy metabolism control, ranging from food intake and satiety mechanisms to adipogenesis, white adipocyte browning and brown adipocyte activation [25,61,62,63,64]. In the present study, we assessed the effects of a 12 weeks dietary supplementation of CAE (equivalent to 4 mg capsaicinoids/day, of which 2.4 mg were capsaicin, 1.4 mg were dihydrocapsaicin and 0.2 mg were nordihydrocapsaicin) in reproductive-aged women with overweight and obesity undergoing a calorie-reduced diet (−500 kcal/day) on the gut microbiota and the expanded endocannabinoid system known as the eCBome. These are two major players that control energy homeostasis, independent from their effects on body weight and food intake, and that are disrupted in obesity and other metabolic disorders [32,37]. We report for the first time that CAE inhibited changes in circulating eCBome lipids induced by the caloric restriction in control participants and induced alterations in the gut microbiota in directions that may favor beneficial actions on the metabolic consequences of obesity. Indeed, some eCBome mediators, such as OEA and other non-CB1-activating NAEs, can be considered as biomarkers of metabolic health since their endogenous concentrations in blood or tissues correlate with, among others, increased lipolysis and insulin sensitivity and reduced body fat, dyslipidemia and systemic inflammation, with or without body-weight-reducing interventions [65,66,67], as well as with the dietary intake of the corresponding fatty acids [68].

As mentioned above, capsaicin was shown in experimental models of obesity to affect both endocannabinoid signaling by counteracting CB1 activity, and gut microbiota composition [39,60]. TRPV1 activation by this compound in isolated cells expressing the human recombinant protein was found to lead to the biosynthesis of NAEs, including AEA, OEA and PEA [30]. More recently, TRPV1 activation was also shown to increase MAG levels [69]. Here we observed that CAE counteracted a time-dependent reduction in the circulating levels of NAEs observed in placebo-treated women. In particular, the treatment reversed the reduction in the levels of the anorectic and lipolysis-inducing and energy-expenditure-inducing mediator OEA, which acts via the activation of TRPV1, as well as of the pro-lipolytic peroxisome proliferator-activated receptor α (PPARα) and, possibly, the GLP-1-release stimulating GPCR, GPR119 [70,71,72], with no effect on AEA nor on the other CB1-activating mediator, 2-AG. CAE also counteracted the reduction of the levels of LEA and 2-OG, two other endogenous TRPV1 and GPR119 agonists [73,74], and of the anti-inflammatory NAEs, namely, DHEA and DPEA [74,75,76]. The decrease in non-CB1 activating NAEs by caloric restriction is likely to represent an adaptive, negative feedback mechanism, and was observed for OEA also in the small intestine of rats following food deprivation [77]. By being partly in agreement with the aforementioned in vitro studies showing that TRPV1 activation by capsaicin stimulates NAE biosynthesis [30,69], our present findings may suggest that, in the subset of participants of our cohort in whom the plasma eCBome was measured, capsaicinoids, administered as CAE, were acting via TRPV1 channels. More importantly, these findings indicate that CAE positively affected the peripheral levels of metabolically beneficial NAEs without changing those of CB1-activating, and hence potentially obesity-exacerbating, endocannabinoids, i.e., AEA and 2-AG.

Several studies have linked the anti-obesity effects of capsaicin to modulation of the gut microbiome, including increasing the abundance of the “anti-obesity” species *Akkermansia muciniphila* [40,60,78]. Results from faecal microbiota transplantation, as well as antibiotics treatments experiments, suggest that capsaicin-induced anti-metabolic endotoxemia effects were at least partly dependent on modulation of the microbiome in mice [39]. CAE also produced some effects on gut microbiota composition in our study by both counteracting the increased diversity observed with caloric restriction only and affecting the relative abundance of some taxa. In particular, it tended to increase the *Bacteroidetes* phylum, increase the *Clostridia* class and increase the *Flavonifractor* genus. While the former two might exert beneficial metabolic effects, the increase in *Flavonifractor* might relate to the capability of this genus to metabolize flavonoids and, in particular, curcumin, with which capsaicin has structural homology. Accordingly, dietary curcumin or curcumin-containing turmeric were also shown to increase the abundance of *Flavonifractor sp.* in human fecal microbiota in vitro [79] or in vivo [80]. Species belonging to this genus, such as *F. plautii*, were suggested to be decreased in obesity/increased in leanness [81] and play anti-inflammatory actions [82]. Interestingly, it appears that an individual’s gut microbiota composition can modulate the response to capsaicin. In a trial involving healthy adults, it was observed that a broad separation of participants into *Bacteroides* and *Prevotella* enterotypes similarly stratified the participants into either strong or weak capsaicin responders, respectively, with respect to capsaicin-induced increases in *Faecalibacterium* abundance and serum incretin (GIP and GLP-1) levels [83]. While we did observe a trend toward an increase in *Faecalibacterium* abundance in response to CAE (data not shown; *p* = 0.14, V1 vs. V7 CAE), the results may have been confounded by the fact that baseline *Faecalibacterium* abundance was significantly lower in the CAE V1 group as compared to the placebo V1 group (*p* = 0.014). We previously proposed that this difference between enterotypes may be due to microbiome-mediated modulation of TRPV1 responsiveness to capsaicin [37], since it was shown that the probiotic *Lactobacillus reuteri* can inhibit TRPV1 activity in mesenteric neurons [84]. Thus, the relatively small changes in the gut microbiome that we observed here may in part be due to the participants harboring capsaicin-resistant microbiomes, which may be generalizable and explain why capsaicin effects on energy expenditure were reported to be blunted in obese as compared to lean individuals [57]. Nevertheless, we were expecting to see stronger effects of CAE on fecal microbiota composition. It is possible that some effects, as mentioned above, were masked by the fact that the relative abundance of some taxa were different at the baseline (V1) in the two control groups, which was a limitation of this study. However, in agreement with previous studies showing the existence of a bilateral communication between the host eCBome and the gut microbiome [85], we did find several correlations between the eCBome mediators whose concentrations in the plasma were modified by CAE and numerous gut bacterial taxa, also at the genus level. Of these, some seemed to be particularly relevant to the potential beneficial metabolic effects of capsaicin. The most obvious of these was undoubtedly *Akkermansia*, which positively correlated with PEA levels (as did the *Akkermansiaceae* family). *Akkermansia* abundance is decreased in obesity and a recent clinical trial showed that supplementation, especially with heat-inactivated *Akkermansia*, improved metabolic parameters in obese participants, including decreasing hepatic inflammation markers [86]. Interestingly, PEA is a potent anti-inflammatory compound with reported metabolic benefits in models of obesity, including a reduction in high-fat diet-induced liver inflammation [87,88]. In contrast, *Enterobacteriaceae*, a lipopolysaccharide-producing family that is associated with obesity and metabolic endotoxemia [89], was also positively correlated with PEA. *Flavonifractor*, the only genus that was found to be specifically upregulated by CAE in our study, was positively associated with levels of the GPR119 agonist 2-OG (see above). *Flavonifractor* was found to be associated with non-obese as compared to obese individuals and, when provided to mice on a high-fat diet, it attenuated adipose tissue inflammation [81,82]. Similarly, *Parabacteroides* and *Dialister* were also correlated with 2-OG, and different species of the former were tied to the beneficial metabolic effects of fiber and to ameliorate diet-induced metabolic dysfunction [90,91], while the latter was associated with improved insulin sensitivity [92], though paradoxically also with resistance to weight loss in response to a volumetric diet [93]. Taken together, it is clear that the assessment of the significance of these correlations will require further investigation in order to understand whether they have any causal effects on obesity and associated complications.

The CAE-encapsulating technology used here allowed for a controlled release that delivered effective levels of capsaicinoids in the small intestine without the stomach upset that may result from unprotected extracts of red chili peppers. Although we did not aim to compare the difference between such technology and capsaicinoids ingested as supplements with some specific foods, we believe that it is possible that such difference might exist. In particular, capsule release in the small intestine may bypass mouth- and stomach-specific activation of TRPV1 and modify the sensory detection of taste differences in the mouth.

## 5. Conclusions

In summary, in the present exploratory clinical study, the oral administration of CAE to reproductive-aged women living with overweight and obesity produced important changes in the plasma eCBome, which may be indicative of potential beneficial effects at the level of hyperphagia (through increased levels of an anorectic factor, such as OEA), insulin sensitivity (through increases of endogenous agonists of GPR119) and inflammation (through the increase of anti-inflammatory mediators like DPEA and DHEA). However, while we did not see any significant correlation between eCBome mediators and the appetite and satiety measures, here we also did not assess measures of insulin sensitivity or chronic inflammation. This, together with the relatively small number of participants from whom we were allowed to sample blood and stools for eCBome and gut microbiome measurements, represent limitations of this study. Nevertheless, we suggest that some of the observed effects of CAE on eCBome mediators and the gut microbiome, the latter of which probably also represents an attempt of the gut microbial ecosystem to deal with capsaicin and its metabolism, may contribute to potential metabolically beneficial effects of these natural products. In agreement with this hypothesis, in the subset of patients that underwent plasma eCBome and fecal microbiota assessments, CAE performed significantly better than the placebo at reducing fat mass. The finding of strong correlations between several eCBome mediators and fecal microbiota taxa should also be considered in this context and reinforces the hypothesis of the existence of a connection between these two metabolically relevant signaling systems in the framework of overweight and obesity.

## Figures and Tables

**Table 1:** Composition of the OmniActive Health Technologies CAE and placebo capsules.

	OmniActive Health Technologies CAE Capsules	Placebo Capsules
**Medicinal ingredient**	*Capsicum annuum* extract (100 mg)	Corn starch (230 mg)
**Extract (per capsule)**	15:1 DHE 1.5 mg (dry herb)	
**Potency (per capsule)**	0.7% Dihydrocapsaicin, 0.1% nordihydrocapsaicin and 1.2% capsaicin (2% capsaicinoids)	
**Non-medicinal ingredients**	Cellulose gum, hypromellose	Cellulose gum, hypromellose
**Pharmaceutical glaze**	Sucrose	Sucrose

**Table 2:** Response to weight loss and CAE interventions. The *t*-test (paired where appropriate) *p*-values were computed for selected anthropometric parameters (BMI, body weight, waist circumference, fat mass percentage, fat-free mass); ns: not statistically significant.

Variable	Treatment	V1		V7		*p*-Value	*p*-Value	*p*-Value
		Mean	SE	Mean	SE	Treatment	Time	Interaction
**BMI**	**Placebo**	29.47	0.7	28.78	0.7	ns	0.0001	ns
	**CAE**	28.99	0.7	27.84	0.8			
**Body weight** **(kg)**	**Placebo**	81.2	2.1	78.7	2.1	0.1	<0.0001	0.1
	**CAE**	76.1	2.4	73.2	2.4			
**Waist** **Circumference (cm)**	**Placebo**	91.8	1.8	89.8	1.8	ns	<0.0001	ns
	**CAE**	88.4	2.1	85.9	2.1			
**Fat mass (%)**	**Placebo**	40.6	0.9	39.3	0.9	ns	<0.0001	ns
	**CAE**	39.7	1.1	38.1	1.1			
**Fat-free mass (kg)**	**Placebo**	45.5	1.0	45.2	1.0	0.08	0.02	ns (0.08)
	**CAE**	43.1	1.1	42.5	1.1			

**Table 3:** Effects of weight loss and CAE on pre- and postprandial appetite sensation, as assessed using the visual analogous scale. Desire to eat (DE), hunger (Hun), satiety (Sat) and prospective food consumption (PFC) were evaluated before (V1) and after the interventions (V7). The area under the curve (AUC) was measured for the first hour (60 min) and the subsequent two hours (60–180 min) following the breakfast test; ns: not statistically significant.

Variable	Treatment	V1		V7		*p*-Value	*p*-Value	*p*-Value
		Mean	SE	Mean	SE	Treatment	Time	Interaction
**DE_60 (mm)**	**Placebo**	1576	277	1792	281	ns	0.1	ns
	**CAE**	1265	311	1678	311			
**DE_180 (mm)**	**Placebo**	5308	551	6253	560	0.07	0.02	ns
	**CAE**	3842	619	5017	619			
**Hun_AUC_60**	**Placebo**	1246	262	1775	267	ns	0.08	ns
	**CAE**	1122	294	1401	294			
**Hun_AUC60_180**	**Placebo**	4800	573	5832	583	ns	0.04	ns
	**CAE**	4225	644	5023	644			
**Sat_AUC_60**	**Placebo**	6536	299	6353	304	ns	ns	ns
	**CAE**	6596	336	6762	336			
**Sat_AUC60_180**	**Placebo**	10,698	651	10,028	659	ns	ns	ns
	**CAE**	10,846	732	11,116	732			
**PFC_AUC_60**	**Placebo**	2032	293	2190	297	ns	ns	ns
	**CAE**	1786	329	1941	329			
**PFC_AUC60_180**	**Placebo**	6049	539	6818	548	ns	0.04	ns
	**CAE**	5102	606	6026	606			

**Table 4:** Effects of weight loss and CAE supplementation on feelings of hunger, as measured with the Three-Factor Eating Questionnaire (TFEQ) before (V1) and after the interventions (V7). Note that this table includes the TFEQ values from all individuals that completed the trial; ns: not statistically significant.

Variable	Treatment	V1		V7		*p*-Value	*p*-Value	*p*-Value
		Mean	SE	Mean	SE	Treatment	Time	Interaction
**TFEQ_hunger**	**Placebo**	5.96	0.64	4.17	0.64	ns	ns	0.09
	**CAE**	5.27	0.72	5.75	0.73			

**Table 5:** Effects of weight loss and CAE supplementation on appetite sensations in self-administered VAS-derived and Three-Factor Eating Questionnaire (TFEQ) measured variables before (V1) and after the interventions (V7). DQuaN, pre-meal evaluation of the amount of food that could be eaten; BEnvM, pre-meal desire to eat; BSenF, feeling of hunger; TFEQ-HUN, habitual feelings of hunger; TFEQ-HEL, feelings of hunger arising from external sources; TFEQ-HIL, feelings of hunger arising from internal sources. This table represents the measurements made in the subset of participants that had both their plasma eCBome and fecal microbiota composition characterized. Effects of the Capsimax supplementation on appetite sensations were measured before (0) and 30 or 180 min following breakfast; ns: not statistically significant.

Variable	Treatment	V1		V7		*p*-Value	*p*-Value	*p*-Value
		Mean	SE	Mean	SE	Treatment	Time	Interaction
**DQuaN0**	**Placebo**	28.1	5.0	104.6	4.9	ns	<0.001	ns
	**CAE**	26.6	5.9	99.8	4.7	ns	<0.001	ns
**DQuaN180**	**Placebo**	78.3	6.8	80.5	4.8	ns	ns	ns
	**CAE**	70.6	6.8	76.7	5.2	ns	ns	ns
**BEnvM_30**	**Placebo**	85.9	6.9	99.9	3.6	ns	ns	ns
	**CAE**	82.6	7.2	84.5	6.7	ns	ns	ns
**BEnvM180**	**Placebo**	39.2	5.9	40.5	4.7	ns	ns	ns
	**CAE**	43.5	6.5	42.2	6.8	ns	ns	ns
**BSenF_30**	**Placebo**	81.3	7.1	94.4	5.6	ns	ns	ns
	**CAE**	86.7	6.9	80.8	6.0	ns	ns	ns
**BSenF180**	**Placebo**	34.2	5.1	42.7	4.5	ns	ns	ns
	**CAE**	47.2	6.6	39.7	5.4	ns	ns	ns
**TFEQ-HUN**	**Placebo**	5.5	0.7	4.2	0.5	ns	0.04	ns
	**CAE**	5.7	0.7	5.9	0.9	ns	ns	ns
**TFEQ-HIL**	**Placebo**	1.8	0.4	1.3	0.3	ns	0.05	ns
	**CAE**	1.8	0.4	1.9	0.5	ns	ns	ns
**TFEQ-HEL**	**Placebo**	2.4	0.3	1.8	0.3	ns	ns	ns
	**CAE**	2.7	0.3	2.7	0.4	ns	ns	ns

## Data Availability

Data is contained within the article or Supplementary Materials.

## Supplementary Materials

The following are available online at https://www.mdpi.com/article/10.3390/biomedicines9091246/s1. Table S1: Reported symptoms and inventory of collected samples, Table S2: List of the lipid mediators used as deuterated internal standards for LC/MS-MS analyses, Figure S1: Effect of Capsimax and caloric restriction on relative abundance of fecal bacterial genera, Figure S2: Correlation between fecal microbiota taxa at phylum (A), classed (B) and genera (C) levels and BMI, waist circumference, fat mass (FM) and fat-free mass (FFM), Figure S3: Correlations between the plasma levels of eCBome mediators and BMI, waist circumference, fat mass (FM) and fat-free mass (FFM).
