# Supplementary material for: Oral Capsaicinoid Administration Alters the Plasma Endocannabinoidome and Fecal Microbiota of Reproductive-Aged Women Living with Overweight and Obesity
Source: Biomedicines. 2021 Sep 17;9(9):1246. doi: 10.3390/biomedicines9091246 (PMC8471891; doi:10.3390/biomedicines9091246)
Supplement: Supplementary file 1 [file biomedicines-09-01246-s001.zip › biomedicines-1342932-supplementary.pdf]

Table S1: Reported symptoms and inventory of collected samples.

Number	Age	Reported Symptoms	Completed trial (C) / Dropout (DO)	Blood samples		Stool samples	
				V1	V7	V1	V7
**CAE-001**	23	Little	C	-	-	-	-
**CAE-002**	20	Little. Vomiting. Transient constipation	C	-	-	-	-
**CAE-003**	40	At first: Cramps and diarrhea. then constipation. stomach ache	C	-	-	-	-
**Pla-004**	39	At first: Headache. heartburn. stools more frequent and loose	C	-	-	-	-
**Pla-005**	19	None	C	-	-	-	-
**CAE-006**	27	None	C	-	-	-	-
**Pla-007**	34	None	C	-	-	-	-
**CAE-008**	31	At first: Stools more frequent. headache. heartache	C	-	-	-	-
**Pla-009**	23	None	DO	-	-	-	-
**CAE-010**	44	Burning sensation in the rectum a few days. diarrhea. nausea. stomach ache. vomiting	C	-	-	-	-
**CAE-011**	27	V5 and up: nausea. sweating. intestinal burning	C	-	-	-	-
**Pla-012**	40	At first: Sensation of acidity in the stomach 1 time	C	-	-	-	-
**CAE-013**	44	None	C	-	-	-	-
**CAE-014**	24	At first: Bowel pain. lower abmen. poor appetite	DO	-	-	-	-
**Pla-015**	21	None	C	-	-	-	-
**Pla-016**	28	None	C	-	-	-	-
**Pla-017**	34	V3 and V5: heartburn	C	-	-	-	-
**Pla-018**	40	None	DO	-	-	-	-
**Pla-019**	41	None	C	x	x	x	x
**CAE-020**	25	Intestine and stomach level bar. cramps.<br>V3: Sweating. V5-6: Belly ache	C	x	x	-	-
**Pla-022**	34	At first: Stool more frequent. then constipation. nausea	C	x	x	-	-
**CAE-023**	26	Heat and sweats. more appetite. hot flashes. gas and bloating	C	x	x	-	-
**Pla-024**	25	None	DO	-	-	-	-
**CAE-025**	21	Burning sensation. stomach pain. vomiting in PM. previous stomach ache	C	x	x	-	-
**Pla-026**	50	None	C	x	x	x	x
**CAE-027**	47	V3: Red urine. Migraine	DO	x	-	x	-
**CAE-028**	28	Headache. burning and stomach pain. vomiting. pain	DO	x	-	x	-

		and burning during bowel movements					
**Pla-029**	21	None	C	x	x	x	x
**Pla-030**	25	None	C	-	-	-	-
**CAE-031**	35	None	C	x	x	x	x
**Pla-032**	29	At first: Diarrhea	DO	x	-	x	-
**CAE-033**	26	At first: Gastro-oesophageal reflux. flatulence. diarrhea	C	x	x	x	x
**CAE-034**	26	None	DO	x	-	-	-
**Pla-035**	26	At first: Dry mouth. flatulence and night sweats	C	x	x	x	x
**Pla-036**	21	V3: no symptoms but nausea if not eating within hours of taking	DO	x	-	x	-
**CAE-037**	47	Gurgling the first days	DO	x	-	-	-
**CAE-038**	22	None	C	-	-	x	-
**Pla-039**	26	None	C	x	x	-	-
**CAE-040**	30	Diarrhea and heartburn. headache. vomiting	DO	-	-	x	-
**Pla-041**	25	At first: Dizziness	C	x	x	x	x
**Pla-042**	25	V4: Bloating and stomachaches	C	x	x	x	x
**CAE-043**	22	Morning canceled	DO	-	-	x	-
**Pla-044**	23	V2: Tremors and heartaches	C	-	-	x	x
**CAE-045**	23	Stomach burns. spasms. reflux. bloating	DO	-	-	-	-
**CAE-046**	24	Heartache. vomiting. stomach cramps. loose stools	C	x	-	-	-
**Pla-047**	28	V3: Diarrhea and nausea	C	x	x	x	x
**CAE-048**	38	Constipation. blood in the stools. stinging and burning stools	C	x	x	x	x
**Pla-049**	25	None	C	x	x	x	x
**CAE-050**	22	Constipation and burning during evacuation. hard stools. diarrhea. stomachaches. headache. dizziness. intense migraine	DO	x	-	-	-
**Pla-051**	35	None except V6 stomach pains ++ after 6 oranges	C	x	x	x	x
**Pla-052**	36	None	C	-	-	x	x
**CAE-053**	24	Discomfort. vomiting. loose stools. cramps. abdominal pain. stinging stools. diarrhea. nausea and stomach discomfort	C	x	x	x	x
**Pla-055**	33	At first: Hot flashes. V6: mild heartburn	C	x	x	-	-
**CAE-056**	45	V4: loss of appetite. less hunger and less sugar rage	DO	x	-	-	-
**Pla-057**	20	At first: V2-3: Headache. 1 episode of vomiting	C	x	x	x	-

**Pla-058**	29	None	C	-	-	x	x
**CAE-059**	27	At first: Bellyache. loose stools. pressure drop	C	x	x	-	-
**CAE-060**	31	Vomiting. stomach pain and gas ++. heartburn. burping. bloating	C	x	x	x	x
**CAE-061**	20	Sudden epigastric abdominal pain 10/10. loss of consciousness. hospital transport by ambulance	DO	-	-	-	-
**CAE-062**	24	Bellyache. stomach. burning and swelling in the stomach. gas and burps. diarrhea	C	x	x	x	x
**Pla-063**	19	Gurgling	DO	-	-	-	-

Table S2: List of the lipid mediators used as deuterated internal standards for LC/MS-MS analyses

Abbreviation	Internal standards utilized
**2-AG**	d5-Mono-arachidonoyl-glycerol 1(3) and 2 isomens
**AA**	d8-Arachidonic acid
**AEA**	d4-Anandamide
**Arachidonoyl Glycine**	d8-*N*-Arachidonoyl Glycine
**Arachidonoyl Serotonin**	*N*-Arachidonoyl Serotonin
**DHA**	d5-Docosahexaenoic acid
**DHEA**	d4-*N*-docosahexaenoyl-ethanolamine
**DPA**	d5-Docosapentaenoic acid
**2-DPG (n-3)**	d5-Mono-docosapentaenoyl-glycerol 1(3) and 2 isomers
**EPA**	d5-Eicosapentaenoic acid
**2-EPG**	d5-Mono-eicosapentaenoyl-glycerol 1(3) and 2 isomers
**EPEA**	d4-*N*-eicosapentaenoyl-ethanolamine
**LEA**	d4-*N*-linoleoyl-ethanolamine
**2-LG**	d5-Mono-linoleoyl-glycerol 1(3) and 2 isomers
**N-Oleyl-L-Serine**	*N*-Oleyl-L-Serine
**2-OG**	2-oleoyl-glycerol
**Oleoyl Serotonin**	d17-*N*-oleoyl-serotonin
**OEA**	d4-*N*-oleoyl-ethanolamine
**PEA**	d4-*N*-palmitoyl-ethanolamine
**2-PG**	Mono-palmitoyl-glycerol 1(3) and 2 isomers
**PGE2**	d4-Prostaglandin E2
**PGE2-EA**	d4-Prostaglandin E2 ethanolamide (prostamide E2)
**PGE2-G**	d5-Prostaglandin E2 glycerol
**PGF2α-EA**	Prostaglandin F2α ethanolamide (prostamide F2α)
**Stearoyl-EA**	d3-*N*-stearoyl-ethanolamine

Legends to Supplementary Figures:

Figure S1: Effect of Capsimax and caloric restriction on relative abundance of fecal bacterial genera of 15 participants (9 placebo and 6 Capsimax) overweight or obese reproductive aged-women, before (Visit 1 [V1]) and after (Visit 7 [V7]) the interventions.

Figure S2: Correlation between fecal microbiota taxa at phylum (A), classed (B) and genera (C) levels and BMI, waist circumference, fat mass (FM) and fat-free mass (FFM) of 15 participants (9 placebo and 6 Capsimax) overweight or obese reproductive aged-women. The correlation analysis were performed using repeated measures correlation tests (rmcorr package). Only correlations coefficients with p-value < 0.05 are displayed.

Figure S3: Correlations between the plasma levels of eCBome mediators and BMI, waist circumference, fat mass (FM) and fat-free mass (FFM) of 15 participants (9 placebo and 6 Capsimax) overweight or obese reproductive aged-women. The correlation analysis was performed using repeated measures correlation tests (rmcorr package). Only correlations coefficients with p-value < 0.05 are displayed. OEA, *N*-oleoyl-ethanolamine; LEA, *N*-linoleoyl-ethanolamine; DPEA, *N*-docosapentaenoyl-ethanolamine; 1/2-OG, 1/2-oleoyl-glycerol; DHA, docosahexaenoic acid; DHEA, *N*-docosahexaenoyl-ethanolamine; PEA, *N*-palmitoyl-ethanolamine.

Figure S1

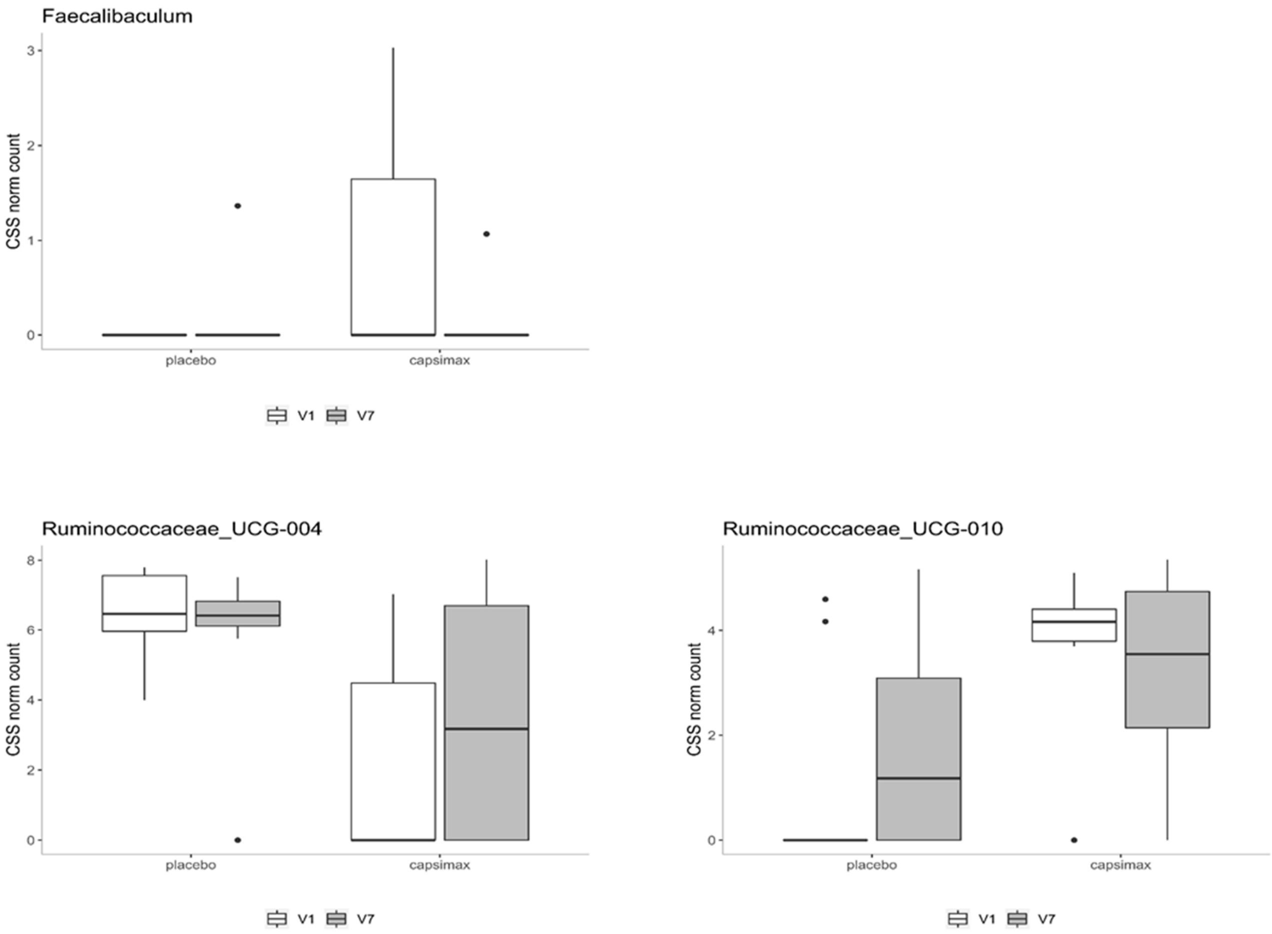
Faecalibaculum
CSS norm count
0
1
2
3
placebo
capsimax
V1
V7
Ruminococcaceae_UCG-004
CSS norm count
0
2
4
6
8
placebo
capsimax
V1
V7
Ruminococcaceae_UCG-010
CSS norm count
0
2
4
placebo
capsimax
V1
V7

Figure S2

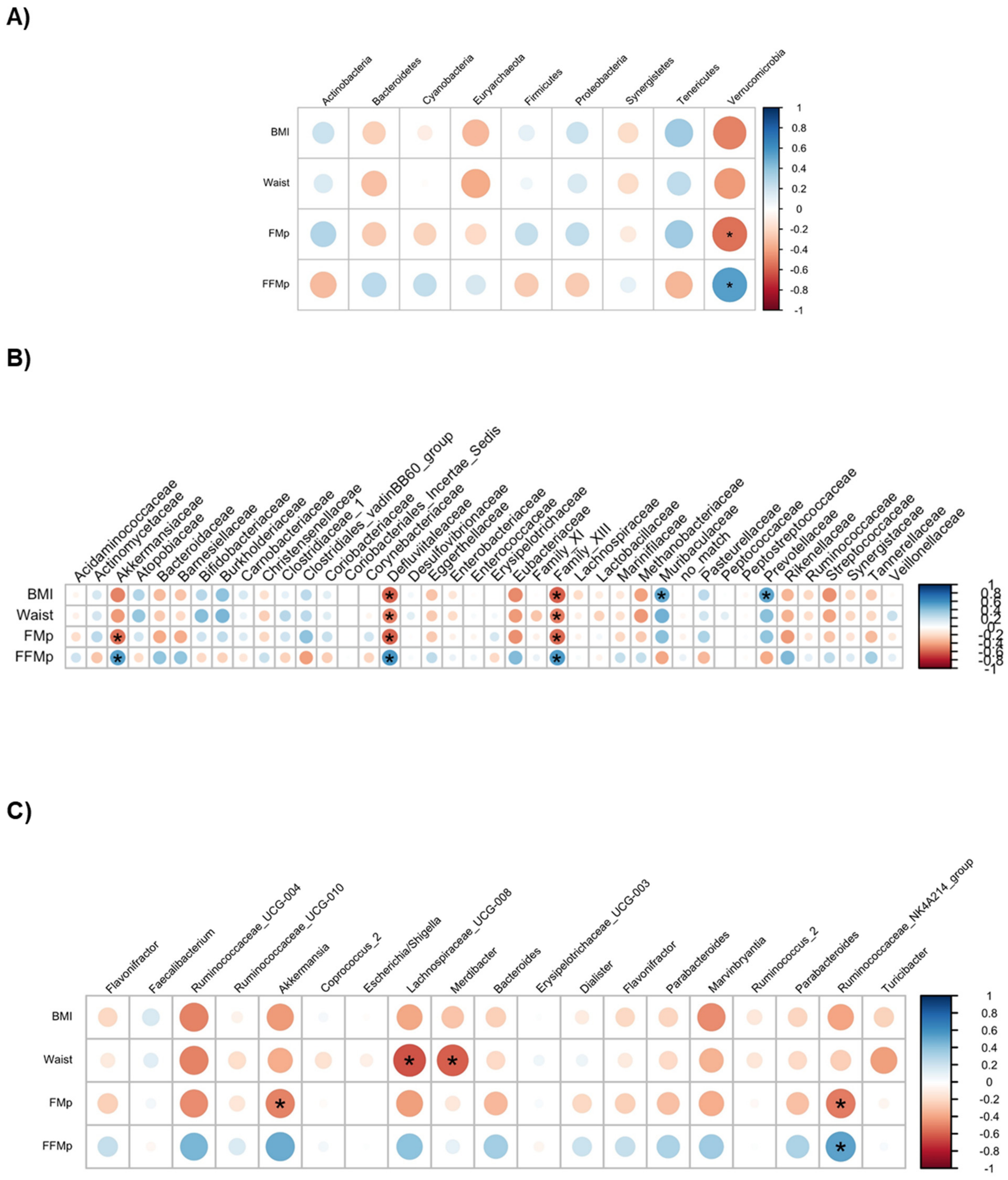
A)
Actinobacteria
Bacteroidetes
Cyanobacteria
Euryarchaeota
Firmicutes
Proteobacteria
Synergistetes
Tenericutes
Verrucomicrobia
BMI
Waist
FMp
FFMp
1
0.8
0.6
0.4
0.2
0
-0.2
-0.4
-0.6
-0.8
-1
B)
Acidaminococcaceae
Actinomycetaceae
Akkermansiaceae
Atopobiaceae
Bacteroidaceae
Barnesiellaceae
Bifidobacteriaceae
Burkholderiaceae
Carnobacteriaceae
Christensenellaceae
Clostridiaceae_1
Clostridiales_vadinBB60_group
Coriobacteriaceae
Coriobacteriales_Incertae_Sedis
Corynebacteriaceae
Defluviitaleaceae
Desulfovibrionaceae
Eggerthellaceae
Enterobacteriaceae
Enterococcaceae
Erysipelotrichaceae
Eubacteriaceae
Family_XI
Family_XIII
Lachnospiraceae
Lactobacillaceae
Marinifilaceae
Methanobacteriaceae
Muribaculaceae
no_match
Pasteurellaceae
Peptococcaceae
Peptostreptococcaceae
Prevotellaceae
Rikenellaceae
Ruminococcaceae
Streptococcaceae
Synergistaceae
Tannerellaceae
Veillonellaceae
BMI
Waist
FMp
FFMp
C)
Flavonifractor
Faecalibacterium
Ruminococcaceae_UCG-004
Ruminococcaceae_UCG-010
Akkermansia
Coprococcus_2
Escherichia/Shigella
Lachnospiraceae_UCG-008
Merdibacter
Bacteroides
Erysipelotrichaceae_UCG-003
Dialister
Flavonifractor
Parabacteroides
Marvinbryantia
Ruminococcus_2
Parabacteroides
Ruminococcaceae_NK4A214_group
Turicibacter
BMI
Waist
FMp
FFMp
1
0.8
0.6
0.4
0.2
0
-0.2
-0.4
-0.6
-0.8
-1

Figure S3

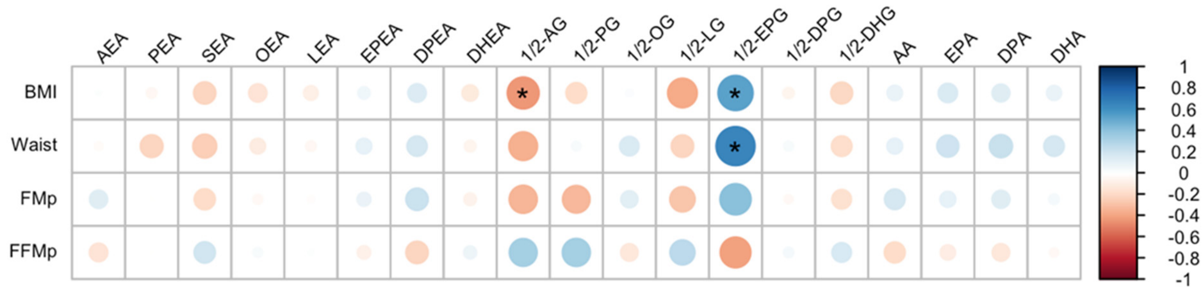
AEA
PEA
SEA
OEA
LEA
EPEA
DPEA
DHEA
1/2-AG
1/2-PG
1/2-OG
1/2-LG
1/2-EPG
1/2-DPG
1/2-DHG
AA
EPA
DPA
DHA
BMI
Waist
FMp
FFMp
*
*
*
1
0.8
0.6
0.4
0.2
0
-0.2
-0.4
-0.6
-0.8
-1
